# Through Silicon MEMS Inspection with a Near-Infrared Laser Scanning Setup

**DOI:** 10.3390/s25154627

**Published:** 2025-07-25

**Authors:** Manuel J. L. F. Rodrigues, Inês S. Garcia, Joana D. Santos, Filipa C. Mota, Filipe S. Alves, Diogo E. Aguiam

**Affiliations:** International Iberian Nanotechnology Laboratory, Avenida Mestre José Veiga, 4715-330 Braga, Portugal; ines.garcia@inl.int (I.S.G.); joana.santos@inl.int (J.D.S.); filipa.mota@inl.int (F.C.M.); filipe.alves@inl.int (F.S.A.)

**Keywords:** MEMS, IR Inspection tool, nanometrology

## Abstract

The inspection of encapsulated MEMS devices typically relies on destructive methods which compromise the structural integrity of samples. In this work, we present the concept and preliminary experimental validation of a laser scanning setup to non-destructively inspect silicon-encapsulated microstructures by measuring small variations of transmitted light intensity in the near-infrared spectrum. This method does not require any particular sample preparation or damage, and it is based on the higher degree of transparency of silicon in the near-infrared and the transmission contrast resulting from the Fresnel reflections observed at the interfaces between the different materials of the MEMS device layers. We characterise the small feature resolving performance of the laser scanning setup using standard targets, and experimentally demonstrate the inspection of a MEMS latching device enclosed within silicon covers, comparing the contrast measurements with theoretical predictions.

## 1. Introduction

Silicon-on-Insulator (SOI) based MEMS device designs typically require bulk micromachining of both the device and handle layer. The former includes features such as springs and anchors, electrostatic actuators and sensing electrodes, while the latter includes the necessary structural supports and open backside cavities. The stacking of multiple micro-structured silicon wafers is often used to combine features in 3D MEMS devices [1]. Non-destructive inspection and characterization of MEMS devices and processes is essential during both the research and development phase and their industrial production. Multiple metrology steps are executed during the fabrication process to ensure that the structure dimensions, such as electrodes and gaps, and deposited or etched material thicknesses, such as metals or oxides, are within the acceptable tolerances for device performance. To ensure reliable integration, the definition of alignment features between front and backside masks and between multiple wafers is required, as well as their validation through inspection [2].

In addition, MEMS devices require proper encapsulation not only to achieve operational robustness but also to enhance their performance [3]. For example, resonant MEMS devices often rely on vacuum packaging to minimize damping losses and achieve a high quality factor. Inertial MEMS sensors may also rely on controlled atmospheres to ensure operational performance [4]. Typical packaging technologies employ ceramic, metal or plastic materials to encapsulate the mechanical structures, and allow for the electrical connections to the electronics, namely using through silicon vias or lateral feedthroughs [5]. Monolithic silicon encapsulation of the MEMS devices on completion of their microfabrication process provides an appealing approach to reduce costs, and to enable optimal performance for devices requiring hermetic sealing solutions [6,7]. However, the inspection of encapsulated devices is made difficult by the typically opaque nature of the packaging materials.

Conventional cross-sectional imaging, such as scanning-electron or atomic force microscopy, on suspended Si-based MEMS structures, requires decapsulation, physical cross-sectioning, and pre-imaging sample preparation before optical and SEM inspection, which may inevitably destroy the device [8]. The possibility to non-destructively inspect and characterize packaged MEMS devices provides an important advantage, especially during the R&D phase of hermetically sealed devices. A non-destructive workflow based on 3D X-ray microscopy or X-ray tomography is able to image the inside of an encapsulated device, without sample preparation, owing to the high penetrability and somewhat non-destructive nature of X-ray inspection, even through different materials [9,10]. These techniques have been demonstrated to reach down to micrometer voxel resolutions in MEMS and through-silicon-via characterization, depending on the trade-off between material absorption, sample thickness, and long scan times [11,12]. However, they rely on dedicated equipments with potentially dangerous ionizing X-ray sources to operate and do not easily allow for the characterization of actuated MEMS devices during their operation [13].

**Figure 1:**
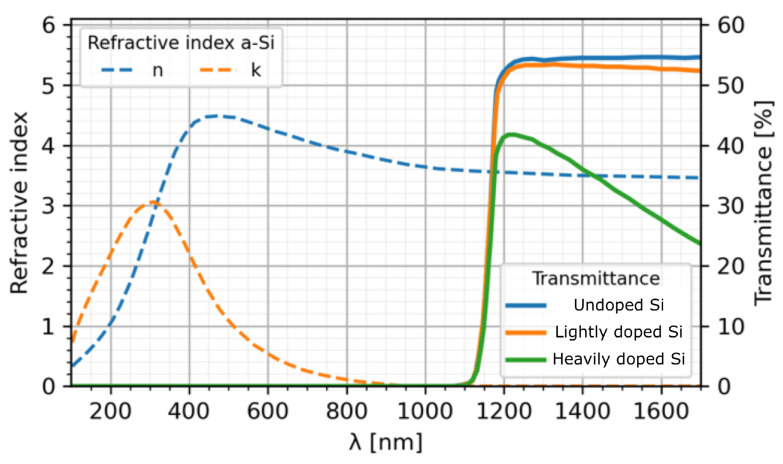
Complex refractive index (n-ik) and optical transmittance including Fresnel losses of silicon for different doping levels, based on measurements presented in [19,20].

On the other hand, infrared microscopy has become an interesting solution for MEMS device characterisation [14,15]. Silicon has an absorption edge at 1.12 eV, becoming opaque in the visible spectrum but mostly transparent in the near-infrared region (λ>1100 nm), as illustrated in Figure 1. The high refractive index of this semiconductor material makes it partially reflective, resulting in high Fresnel losses at the interfaces. In addition, the absorption coefficient within the material increases with doping in the conductive silicon substrates [16]. Taking this optical behaviour into consideration, a light beam in the near-infrared will be able to propagate through SOI-based MEMS devices with transmitted signal attenuations observed at the interfaces between materials and through absorbing materials such as thick doped silicon or metals. Depending on the experimental apparatus, one can resolve small micrometre-sized features within these devices. Low coherence scanning interferometry (CSI) microscopes have been demonstrated to characterize the surface topography of buried silicon MEMS devices, observing electrodes and gaps with lateral resolutions down to 10 µm [17,18]. In CSI setups, a reference mirror is displaced using a piezo actuator relative to the surface under measurement to capture the corresponding correlogram interference patterns. While a large field of view can be acquired using costly infrared-sensitive cameras and magnifying microscope objectives, the CSI method requires post processing and aberration correction to extract surface topography, and the depth measurement is limited by the displacement range of the mirror.

In this work, we present the concept and experimental validation of a near-infrared transmissive laser scanning setup to inspect silicon-encapsulated MEMS devices and resolve features at the micrometric scale by measuring small variations of transmitted intensity. We present the optical scanning system setup and demonstrate its operation in the non-destructive inspection of standard test structure targets and different MEMS devices, reaching lateral resolutions down to 15 µm.

## 2. Optical Inspection System and Methods

**Figure 2:**
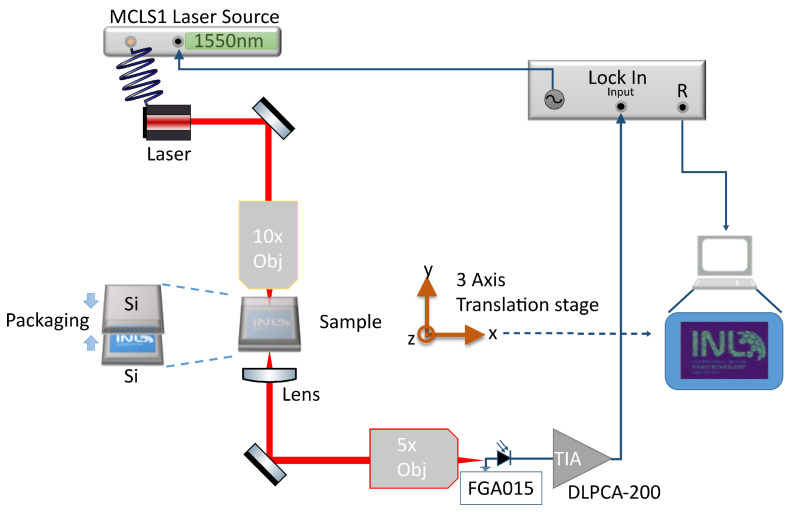
Experimental setup to perform the laser scanning through silicon inspection.

The proposed optical laser scanning inspection setup is illustrated in Figure 2 and is based on confocal transmission microscopy. In this setup, an infrared (IR) laser beam is focused through the sample and the transmission intensity is acquired by a photodetector. The local transmittance is captured as the sample is scanned in-plane, and is attenuated by absorbing materials it encounters, such as metals and highly-doped silicon, as well as the Fresnel losses observed at different material interfaces, such as between silicon-air and silicon-oxide.

The modulated fibre-coupled infrared (λ=1550 nm) laser beam (Thorlabs MCLS1, Thorlabs, Newton, NJ, USA) is focused on a plane in the sample via a 10 × objective 0.25 NA (Thorlabs RMS10X, Thorlabs, Newton, NJ, USA), and the transmitted signal is then collected by a lens with matching NA and collimated to then be focused (Thorlabs RMS4X, Thorlabs, Newton, NJ, USA) on an InGaAs photodiode (Thorlabs FGA015, Thorlabs, Newton, NJ, USA). The sample is scanned in-plane using two orthogonal linear stages (Thorlabs DDS220/M, Thorlabs, Newton, NJ, USA), illuminating different regions of the sample on the focal spot, and vertically using a motorised Lab Jack (Thorlabs L490MZ/M, Thorlabs, Newton, NJ, USA), to adjust the focal plane. The modulated (f = 65.43 kHz) signal is amplified by a transimpedance amplifier (Femto DLCPA-200, FEMTO Messtechnik GmbH, Berlin, Germany) and fed to a lock-in amplifier (SRS SR830, Stanford Research Systems, Sunnyvale, CA, USA), which filters out ambient noise and digitises the acquired intensity and feeds it to the control computer. The scanning and acquisition setup is automated and controlled using Python 3.12 scripts.

Due to the lack of a suitable infrared imaging system in our setup that would allow an operator to quickly visualize the relative positioning of the focused beam ($\omega_{0}=13$ µm) within the sample, we developed an automatic z-scan routine to align the sample for best focus. First, we focus a visible (green) laser pointer, aligned in the same beam path as the IR laser, on the top of Si top cover, hence roughly placing the sample close to the IR beam waist. Then, small 2D regions are consecutively scanned using the IR laser at different vertical positions of the sample and the relative sharpness of the scan is estimated by an algorithm that uses the Sobel–Feldman operator formalism for image processing [21]. This class of gradient operator functions as a convolution filter, and when applied to an image, can be used to calculate the respective sharpness. We begin by convolving the image with a 3 × 3 kernel:

(1) Gxmn=−1−0−1−2−0−2−1−0−1*ImnGymn=−1−2−1−0−0−0−1−2−1*Imn

The resulting image gradient ($G_{mn}$) can then be computed as follows:

(2) $$G_{mn}=\sqrt{G_{x_{mn}}^{2}G_{y_{mn}}^{2}}$$

The sharpness (*S*) is then defined as the sum of all pixel values in the gradient matrix of size m×n:

(3) $$S=\sum_{i=1}^{m}\sum_{j=1}^{n}G_{ij}$$

**Figure 3:**
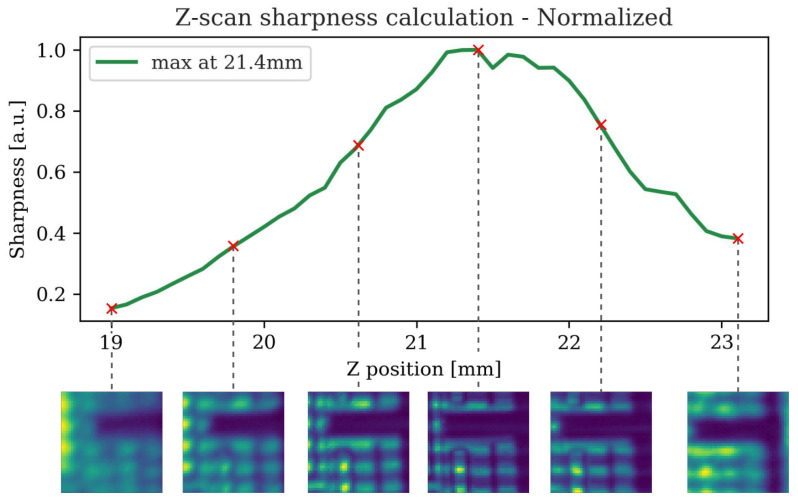
Evolution of the image sharpness during the automatic z-scan to determine the focal plane of the sample.

Figure 3 illustrates one experimental example of the evolution of acquired image sharpness along the z-scan, showing peak sharpness at approximately z ≈ 21.4 mm. This position is subsequently used to scan the larger sample area.

## 3. Experimental Results

### 3.1. Resolving Performance Using Standard Test Target

The performance of the scanning inspection system was first evaluated experimentally using the standard negative USAF 1951 resolution test chart target, which is a known and valuable tool for characterising the resolution of a given optical imaging system. The chart contains repeating line triplet patterns that are organised in elements within groups whose feature size and line spacing are decreasing in size.

**Figure 4:**
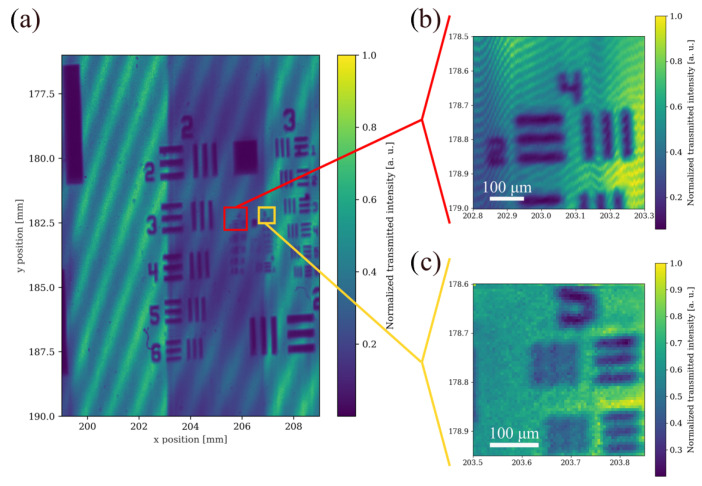
Experimental measurements using the near-infrared laser scanning system of a negative USAF 1951 test chart placed between two polished silicon slabs. (**a**) Large scan with step 10 µm, (**b**) Zoomed scan around group 4, element 2 with step 5 µm. (**c**) Zoomed scan around group 5 element 1 with step 5 µm.

Figure 4 presents the experimental measurements of the test chart using our laser scanning system. To simulate the optical transmittance of an encapsulated MEMS device, the test target was “sandwiched” between two double-side polished silicon slabs, making it opaque in the visible but partially transmissive in the infrared, except for the reflecting metallic resolution structures. The bright regions indicate high transmitted intensity through the silicon slabs, while the dark regions correspond to the reflecting or absorbing metal features of the target. The scanning system is able to easily resolve millimetre-sized features with little distortion. The observed periodic diagonal intensity fringes that appear in the measurements are the result of using a coherent laser source and the consequent multiple-beam interferences that arise due to the small reflections at the multiple material interfaces.

In order to assess the resolution limit of the setup, we scanned the smaller regions around groups 4 and 5 in Figure 4b,c, respectively, using a smaller scan step of 5 µm. We have good 2D resolution for feature sizes that span from tens of millimetres to around a couple of tens of micrometres, such as group 4 element 2, which has a line width of 31 µm. While we can qualitatively still differentiate smaller features in the case of group 5 elements 1 and element 2, which have line widths of 15.63 µm and 13.92 µm, respectively, we are limited by the resolution limit of the current system, which has a beam waist diameter of 26 µm.

### 3.2. Inspection of an Encapsulated MEMS Latching Device

**Figure 5:**
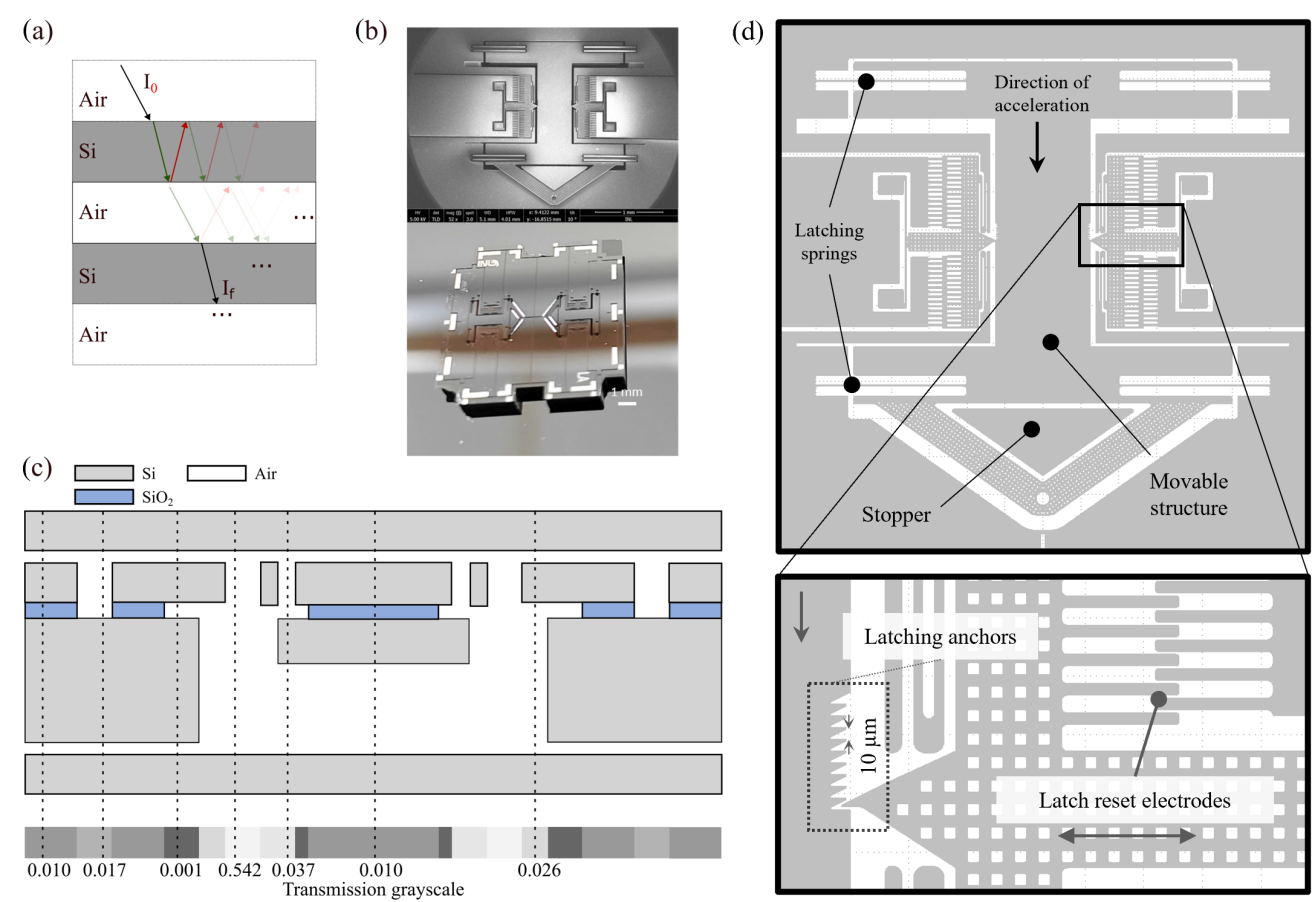
(**a**) Layer stacking of multiple interfaces with several reflections and transmissions. (**b**) SEM (top) and optical (bottom) analysis. (**c**) Latching MEMS multilayer structure with the corresponding intensity transmission coefficients with matching colour bar. (**d**) Schematic and details of the resettable electrostatic latching MEMS device.

Once the performance of the optical inspection system was validated, we used our experimental setup to inspect an encapsulated latching MEMS device, illustrated in Figure 5b,d. Latching mechanisms are commonly used in inertial MEMS switching devices designed to detect an external acceleration above a pre-established threshold. When this acceleration is applied to the movable structure and exceeds the threshold value, it moves towards a fixed contact, closing the electrical circuit. However, during shock impacts, the contact time is usually extremely low, making it difficult to detect an electrical signal arising from this mechanical contact. Therefore, the latching mechanism is employed as a switch that is triggered when the external acceleration surpasses the threshold and remains in that state until a reset is performed. Figure 5d illustrates the details of the latching mechanism. The central movable structure is susceptible to the external downwards acceleration, countered by the latching spring forces. Latching anchors provide multiple stable positions at 10 µm step intervals, which provide linearly-spaced acceleration thresholds. The latching mechanism can be reset through electrostatic actuation, where a voltage signal applied to the electrodes provides a lateral force that dislodges the latching anchor, releasing the movable structure to move back to its initial position. The devices were fabricated in an in-house 4-mask 50 µm-thick SOI wafer bulk micromachinning process, similar to the process described in [22]. This MEMS microfabrication process includes metal patterning for the electrical pads, and patterning of the device layer by DRIE (Deep Reactive Ion Etching), forming features with sizes ranging between 2.25 µm and 80 µm. In addition, an extra handle proof-mass is created by etching an 80 µm-wide path surrounding the mass through DRIE on the handle layer, which is then thinned. Preliminary analysis on the feature size and general operational status of the structures was made beforehand with SEM.

Figure 5c shows the depiction of the stacked multilayer structure of an encapsulated latching MEMS device and the corresponding computed transmittance of the different regions. Assuming normal incidence, as the beam propagates through the heterogeneous multilayer interfaces of the device, transmission losses are incurred due to Fresnel reflections at each interface $r_{i}$ ($i\in N^{0}$) between materials of different refractive index *n*, such as air-Si or Si-SiO_2_, given by [23,24],

(4) $$\begin{array}{c} R_{i}=|r_{i}{|}^{2}={\left|\frac{n_{i+1}-n_{i}}{n_{i+1}+n_{i}}\right|}^{2}\\ T_{i}=\frac{n_{i+1}}{n_{i}}{\left|t_{i}\right|}^{2}=1-R_{i}, \end{array}$$

where $R_{i}$ and $T_{i}$ are the Fresnel reflectance and transmittance at normal incidence. Different contrast levels depend on the stacking and thickness of the layers and to properly study the behaviour of our stacked device to the input light field, we must consider the complex nature of reflection and transmission at successive interfaces and account for losses. For this purpose, we used the transfer matrix method, which relates the forward ($f_{i}$) and backwards ($b_{i}$) wave amplitudes at a certain interface [25,26]:

(5) $$\left(\begin{array}{c} f_{i}\\ b_{i} \end{array}\right)=M_{i}\left(\begin{array}{c} f_{i+1}\\ b_{i+1} \end{array}\right)$$

The transmission\reflection mechanics at the interface between two media is condensed in the matrix:

(6) $$M_{i}=\left(\begin{array}{cc} e^{-i\delta_{i}} & 0\\ 0 & e^{i\delta_{i}} \end{array}\right)\left(\begin{array}{cc} 1 & r_{i,i+1}\\ r_{i,i+1} & 1 \end{array}\right)\frac{1}{t_{i,i+1}}$$

The resulting matrix from the successive interface transitions ($N\in N^{0}$) is then:

(7) $$\tilde{M}=\prod_{j=0}^{N-1}M_{j}=\left(\begin{array}{cc} 1 & r_{0,1}\\ r_{0,1} & 1 \end{array}\right)\frac{1}{t_{0,1}}M_{1}M_{2}\ldots M_{N-1}$$

It is worth mentioning that since the first interface transition occurs between a semi-infinite thickness plane (air) and the first material layer, the respective characteristic matrix has no phase factor included ($e^{i\delta_{i}}$). Considering the exit medium as also being semi-infinite with no backwards component, we can then use the matrix formalism described in Equation (7) and obtain:

(8) $$\left(\begin{array}{c} 1\\ r \end{array}\right)=\tilde{M}\left(\begin{array}{c} t\\ 0 \end{array}\right)=\left(\begin{array}{cc} {\tilde{M}}_{00} & {\tilde{M}}_{01}\\ {\tilde{M}}_{10} & {\tilde{M}}_{11} \end{array}\right)\left(\begin{array}{c} t\\ 0 \end{array}\right)$$

We can obtain the transmission and reflection coefficients,

(9) $$\begin{array}{c} r=\frac{{\tilde{M}}_{10}}{{\tilde{M}}_{00}},\\ t=\frac{1}{{\tilde{M}}_{00}}. \end{array}$$

We computed the expected transmittance and reflectance coefficients for the different regions of the device following Equation (9). For the heterogenous multilayer stacks (Figure 5c), we expect transmittance ranging from 0.001 to 0.54.

**Figure 6:**
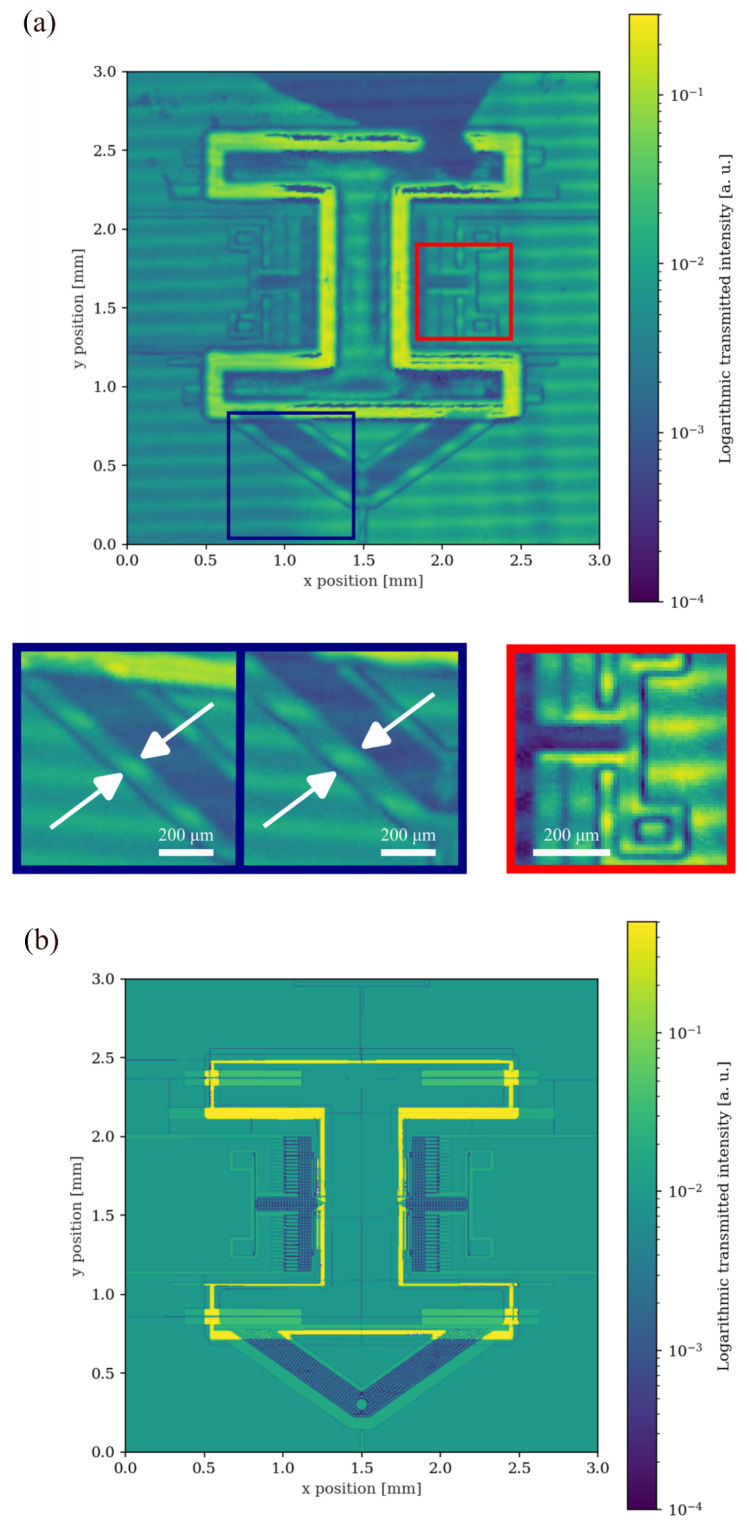
(**a**) Experimental measurement of the encapsulated MEMS device using the infrared scanning inspection setup. Red inset shows the fine details around a structured region. Blue inset shows the difference between the bigger gap in a latched mechanism (left) and the one visible on a non-latched (right). (**b**) Theoretically modelled transmittance map of the same device structures.

The experimental measurement of the encapsulated MEMS device is presented in Figure 6a. The overall structure of the MEMS device can be observed, and most of the critical features on the device layer can be distinguished, although limited by the resolution of the laser spot as characterised previously with the standard target. For example, the latching anchors, however, are not distinguishable due to their reduced size compared to the system resolution. The interference fringes are still observed in this measurement.

**Figure 7:**
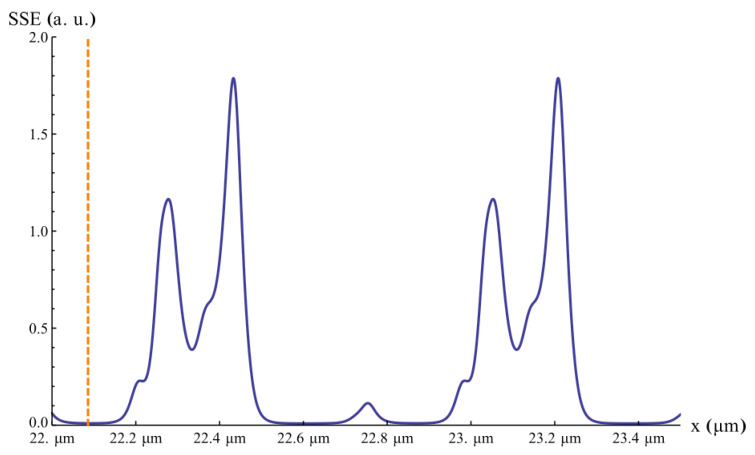
Representation of the evolution of the SSE with the air-gap dimension, represented by *x*. The vertical orange line indicates the minimum value of SSE around 22 µm of air-gap.

The MEMS device uses P-doped silicon in order to achieve high electrical conductivities, with a resistivity in the range of 0.01 to 0.02 Ω/cm. This material differs, in its optical properties, from the intrinsic silicon, resulting a higher internal absorption which, can lead to an overestimation of the transmittance. We compare the measured transmitted intensity of the device with the modelled transmittance map using the respective design masks, as presented in Figure 6b. The theoretical model considers an effective transmission coefficient for silicon, in its interface with air, of about 70%, as a result of setting the index of refraction of silicon to $n_{Si}=3.2$. For SiO_2_ the index of refraction in use is $n_{SiO_{2}}=1.46$ [27]. Differences may arise from the fact that silicon slabs that cover both sides of the device are not perfectly in contact with its surfaces. This can be seen in the fringe effect in Figure 4 and Figure 6a, that arise due to air gaps between the surfaces. To estimate the gap between the surfaces, the sum of squared errors (SSE) between the experimental measured transmission values $T_{i}$ and the theoretical expected value ${\hat{T}}_{i}$, obtained from the model, are calculated as

(10) $$SSE=\sum {\left(\hat{T_{i}}-T_{i}\right)}^{2}.$$

and illustrated in Figure 7, which shows the dependence of the SSE on the air-gap dimension *x*. It is noticeable the periodic behaviour of the multilayer model. This analysis accounts for an average air gap of about 22 µm between the Si covers and the MEMS device, consistent with polymeric adhesive bonding.

**Figure 8:**
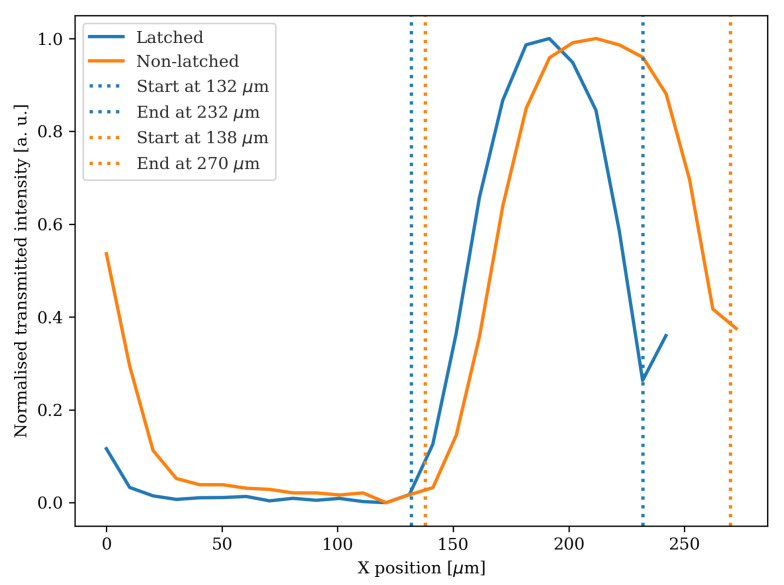
Transmittance profiles for the latched (blue) and non-latched (orange) devices, across the line formed by the arrows in Figure 6a. The vertical dashed lines represent the start and end of the feature under comparison in both cases, indicating an thinner feature size for the latched device.

Having in mind the intended operation of the MEMS device under study, we proceeded to assess the validity of the inspection tool in the analysis of the operational status of the latching mechanism. Since the system resolution limits the observation of the exact latched position near the anchor structures, we measured the gap size, highlighted in the blue inset at Figure 6a, between the movable structure and the outer fixed region. Here, it is visible that in the latched mechanism (left) the clearance in the region indicated by the white arrows is smaller than in the non-latched (right). By analysing the transmittance profiles obtained from these regions, we were able to obtain a difference of around 32 µm in the structural gap between equivalent regions for the latched and non-latched mechanism—Figure 8. This measured gap difference is consistent with a latched device of five steps from the rest position that has a reduction of gap size of 32.9 µm This demonstrates the feasibility of using our non-destructive infrared inspection technique in discussions towards resolving small features in encapsulated MEMS devices.

## 4. Conclusions

This work presents a method to non-destructively inspect silicon-encapsulated MEMS devices using a near-infrared laser scanning setup, without using a costly infrared camera, taking advantage of the high transmittance of silicon in this spectrum range. The small feature resolving performance of the system is first evaluated using a standard USAF 1951 test chart, demonstrating the capabilities of the apparatus to distinguish features down to 15 µm on the focal plane inside the encapsulated device. Then, an encapsulated MEMS latching device was presented and inspected, and the overall device structure and some of the critical features of the device could be observed. We present a model to theoretically approximate the expected transmittance contrast for different multilayer material stacks, which can be adapted to different MEMS processes and multi-wafer 3D integration. We take the transmittance contrast profile to measure the gap sizes of an encapsulated latching device in its latched and non-latched positions, providing a consistent measurement with the expected displacement of the movable structure.

The presented laser scanning setup was demonstrated to map encapsulated MEMS devices. While the lateral resolution of the system can be improved by reducing the beam waist at the focal plane with improved infrared optics, this scanning transmission confocal microscopy measurement is highly susceptible to the scattering features the laser beam encounters, namely sidewall structures and roughness. It is expected that extensive post-processing for aberration correction and deconvolution algorithms are needed to reverse the blurring and reach down to 1 µm resolutions [18,28]. In future applications beyond mapping of encapsulated devices, the system can also be used to position a probing beam on a specific actuated structure inside the device to perform optical interferometric vibrometry measurements, taking advantage of the high bandwidth of the modulated laser and photodetectors, providing a two-in-one mapping and probing functionality with the same hardware.

## Data Availability

The raw data supporting the conclusions of this article will be made available by the authors on request.
